# Evolution of energy and nutrient supply in Zambia (1961–2013) in the context of policy, political, social, economic, and climatic changes

**DOI:** 10.1007/s12571-022-01329-1

**Published:** 2022-11-22

**Authors:** Ndashe Philemon Kapulu, Heather Clark, Simon Manda, Harriet Elizabeth Smith, Caroline Orfila, Jennie I. Macdiarmid

**Affiliations:** 1 School of Food Science and Nutrition, University of Leeds, Leeds, LS2 9JT UK; 2 Zambia Agriculture Research Institute, Ministry of Agriculture, Lusaka, Zambia; 3 The Rowett Institute, University of Aberdeen, Aberdeen, AB25 2DZ UK; 4 School of Politics and International Studies, University of Leeds, Leeds, LS2 9JT UK; 5 School of Earth and Environment, University of Leeds, Leeds, LS2 9JT UK

**Keywords:** Micronutrients, Food balance sheets, Food Security, Nutrition Security, Policy, Zambia

## Abstract

An adequate supply of energy, micronutrients and macronutrients is essential to achieve food and nutrition security to prevent malnutrition. Socio-economic, political, and climatic events, however, can affect the supply of food and nutrients. We assessed country-level supply trends of food and nutrients and their sources within the context of policy changes and political, socio-economic and climatic events from 1961 to 2013 in Zambia. Due to the lack of national food consumption data, food supply data from the FAO food balance sheets, matched to food composition tables, were used to estimate the energy, macronutrient and micronutrient content of 264 food items available to Zambia. We calculated historical nutrient supplies based on demographic characteristics and population-level dietary requirements. Results showed that Zambia was nutrition insecure from 1961 to 2013 for key micronutrients vitamin A, folate, riboflavin, vitamin B12, calcium, zinc, iron, and energy-deficient from the late-1980s. The diet has not substantially changed over time, with maize being the dominant food source. However, refined energy-dense food has steadily increased in the diet coupled with a reduction in fibre. These nutrient supply and dietary pattern trends coincide with specific socio-economic, policy, political, and climatic events from the 1970s to the early-mid 2000s, such as population growth, maize subsidy and crop diversification policies, regime change and drought. This study shows how policy, political and climatic events have been central features shaping nutrient supplies and the consequences for nutrition security. The study provides a context to inform future food policies to improve food and nutrition security.

**Supplementary Information:**

The online version contains supplementary material available at 10.1007/s12571-022-01329-1.

## Introduction

Sub-Saharan Africa (SSA) has faced climate change and political and socio-economic instability in the last six decades (Bain et al., 2013). These have led to food and nutrition insecurity, with insufficient supply of energy, macronutrients and micronutrients leading to chronic malnutrition, especially undernourishment and micronutrient deficiencies. Simultaneously, increased urbanisation has led to a rapid nutrition transition and the emergence of overnutrition, leading many countries in SSA to experience the triple burden of malnutrition (Bain et al., 2013). Furthermore, with a population of 1.1 billion people in 2019, including 40% living in poverty in 2018 (UNPD, 2019; WorldBank, 2019), rapid population growth and poverty have resulted in a lack of food availability and inequalities in food access, increasing chronic undernourishment and undernutrition problems in SSA (Bain et al., 2013). In addition, the prevalence of communicable and non-communicable diseases (NCDs) is increasing among poorer households (Chopra & Darnton-Hill, 2006; FAO, 2009; 2015; UNICEF et al., 2020). At the same time, micronutrient deficiencies, especially zinc, vitamin A, calcium, and iron-deficient anaemia, affect 66%, 53%, 75% and 34% of adult women in eastern and southern Africa, respectively (Correa-Agudelo et al., 2021; Galani et al., 2020). These deficiencies have been attributed to poor diet diversity and pose a severe public health challenge for SSA (Beal et al., 2017).

Food supply at the country level arises from the complex balance of agricultural production and trade and can be affected by many socio-economic, environmental, policy and political factors. Climate change has been shown to affect food production and availability due to disruptions to agricultural production systems (Connolly-Boutin & Smit, 2015). Extreme weather events such as droughts, floods, and extreme temperature fluctuations can increase pests and diseases, collectively impacting agricultural production in countries most vulnerable to climatic changes (Connolly-Boutin & Smit, 2015). The effect has limited the variety and quantity of food available for consumption, impacting food and nutrition security (Bain et al., 2013; Khoury et al., 2014). In the last 60 years, multiple political events, such as regime changes and civil unrest, have occurred throughout SSA, including Zambia. Some countries have made progress towards political stability by transitioning from authoritarian rule to a democratic system, which has affected food production and supply through policy shifts (FAO, 2015). In addition, many SSA countries have experienced economic and policy reforms that have facilitated shifts towards regional integration and trade liberalisation, resulting in changes to food supply. Many countries have opened up to global trade, which was followed by a shift from low to middle-income status and change in dietary pattern (Abrahams et al., 2011; Frayne et al., 2014; Gillespie & van den Bold, 2017a).

While much attention has been paid to the distinct impacts of socio-economic, climatic and political transitions on food supply (FAO et al., 2020; Nelson et al., 2018), the impact of their collective co-occurrence on food and nutrition security is less clear. Food security typically focuses on energy supply and hunger, but this misses many diet-related diseases resulting from a lack of nutrients, particularly micronutrients. In this study, we use Zambia as a case study to explore socio-economic, policy, political and climatic events and trends in food and nutrient supplies relate to food and nutrition security.

Since the 1960s, Zambia has experienced several policy, political, socio-economic and climatic events; for example, a National Food and Nutrition Commission was established in 1967 to coordinate nutritional interventions to improve nutrition security. In addition, several policies and plans aimed at addressing food and nutrition security have been enacted (Harris et al., 2017; Mwanamwenge & Harris, 2017). These are implemented by government ministries including agriculture, health, education, community and social services. However, their impact has not been tracked closely against food and nutrient supplies in the country. Zambia suffers the triple burden of disease and is ranked among the world's most food-insecure countries. Nearly 50% of the Zambian population is undernourished, with 35% of children under five being stunted, 4% wasted, 12% underweight, and 5% overweight in 2016 and 2018 (CSO et al., 2019; Fanzo et al., 2018). Concurrently, about 58% of children and 31% of women have iron deficiency anaemia, and there is also a high prevalence of zinc and vitamin A deficiency among these populations. These deficiencies are associated with low caloric and nutrient intake due to poor diet diversity, with diets comprising mainly cereals (mostly maize) and starchy roots (Harris et al., 2019; Mwanamwenge & Harris, 2017; Fanzo et al., 2018). However, simultaneously in 2016, it was reported that 36.6% of reproductive women and 19.0% of men were overweight, 12.4% and 3.6% were obese, respectively, with 6.7% and 6.5% have type II diabetes, respectively (Fanzo et al., 2018). These illustrate challenges the country faces to improve the population's health and reduce nutrition and health inequalities.

The increasing prevalence of overweight, obesity, and NCD in Zambia, is associated with a nutrition transition the country has been undergoing over the last six decades, with increased consumption of sugars, edible oils and fats, processed foods, and reduced consumption of fibre (Abrahams et al., 2011; Harris et al., 2019; Nnyepi et al., 2015; Popkin et al., 2012). This is also affecting nutrition insecurity as many of these foods are micronutrient poor. Several factors influence nutrition transitions, including growing urbanisation, increased gross domestic production, the growing influence of international supermarkets and fast-food outlets (Hawkes et al., 2017; Laar et al., 2022). In Zambia, previous studies have shown that an increased supply of sugars, fats and oils since the mid-2000s alongside a growing middle class drives this, alongside economic growth and increased cross-country trade, especially food imports (Chisanga & Zulu-Mbata, 2018; Harris et al., 2019; Zhang et al., 2016). However, less is known about how these developments shape the supply of other dietary nutrients, especially micronutrients. Therefore, understanding the supply of micronutrients is critical to inform interventions to address nutrient gaps and safeguard food and nutrition security.

The last known national-level food consumption survey in Zambia was conducted in the early 1970s. Previous food consumption surveys have focused more on specific geographical locations, such as the Urban Food Consumption survey, which does not give a national picture of nutrient supplies (Hichaambwa et al., 2009; NFNC, 2014; CUTS & WFP, 2018). In the absence of national household-level data, food balance sheets (FBS) are often used to assess food supply trends over time (FAO, 2001; FAOSTAT, 2013). Food balance sheets provide crude per capita estimates for food supply, including energy, protein and fat from a wide range of food commodities. For example, Zhang et al. (2016) utilised FAO FBS data to demonstrate an increasing supply of animal source foods, sugars, starchy roots, edible oils, and a decreasing supply of pulses between 1961 and 2013. Meanwhile, Harris et al. (2019) also used FAO FBS (1961 to 2013) to evaluate supply trends for energy, protein, and fat, thus showing an association between diets, nutrition transition, and adverse nutrition outcomes. However, these two studies did not show micronutrient supplies, primarily as these data are not included in FBS, nor did they discuss in the context of policy change, political, climatic or economic events. Our study fills this gap in the literature by mapping micronutrient data to the FBS dataset and studying trends in supply over time. Further, we adjusted the data for household food waste to reduce an overestimate of food available to be eaten.

The novelty of this research explores the co-occurrence of policy, political, socio-economic, and climatic events with nutrition security in Zambia between 1961 and 2013. Precisely, we (i) assess changes in the supply of energy, macronutrients (i.e., protein, fat, carbohydrates and fibre) and micronutrients (i.e., vitamin A, vitamin C, vitamin B12, folate, thiamine, niacin, riboflavin, zinc, iron, and calcium) using FBS from 1961 to 2013 and compare to population-level dietary requirements; (ii) map key social, policy, political, economic and climatic events in Zambia to the supply of dietary nutrients, food and nutrition security across the same period; and (iii) identify lessons based on historical trends to inform the design of appropriate nutrition policy in Zambia. By analysing past trends related to the Zambian food system, the country's future trajectory can be better understood, thus underpinning public health and nutrition interventions.

## Methods

The annual food balance sheets comprising food production, stocks, export and import data for 1961 – 2013 were used (FAOSTAT, 2013). We used data up to 2013 because data beyond this were not available at the time of the study as a new methodology was being developed for the FBS (2014 – 2017), and the continuity of data had not been tested. The FBS provide national-level annual food supply (kg) per capita from total food produced, imported and from stocks minus exports. They do not represent food consumed nor adjust for household food waste.

### Nutrient composition for food commodities

FBS contain data for energy, protein and total fat, which alone do not reflect nutrition security, and therefore, a new dataset with energy, macronutrients and micronutrients was created to match commodity groups. The new energy, fat and protein values were calculated for consistency with the new nutrients included in the dataset and compared to values in the FBS. A summary of key steps followed to estimate Zambia's energy, macronutrient, and macronutrient supplies is shown in the illustration (Online Resource 1).

We followed the following steps to create the dataset:

1. Disaggregation of commodities into food items;
2. Estimating energy, macro-and micro-nutrients by matching FBS food items to West African food composition tables;
3. Adjusting the food items in the commodity group to represent amounts of each food as eaten;
4. By adjusting for household waste;
5. Aggregating the food items back to food commodities with a weighted average, based on the amounts of each food item that is typically eaten.

Nutrient data for the 85 food commodities group were disaggregated into 264 food items (Online Resource 2) and matched with data from the composition tables following the approach described in Macdiarmid et al. (2018). For instance, since FBS only report nutrient values of foods available for human consumption at the commodity level (i.e., ‘maize and products’ or ‘wheat and products’ and not at retail-level (i.e., flour, bread, pasta, or cereal), nutrient data were matched to the disaggregated list of food items based on how foods are consumed at a retail-level. In addition, further adjustments were made for household waste to reflect portions as eaten for each food item, using sub-Saharan Africa regional data as described in the methods here (FAO, 2011; Macdiarmid et al., 2018).

Estimates for energy, macronutrients and micronutrients for each FBS commodity group were primarily derived from the West African Food Composition Tables (FAO, 2012). However, when a food item was not listed in the West African Food Composition Tables or was missing some micronutrient information, values from the United States Department of Agriculture (USDA) nutrient database (USDA, 2019) were used to fill in particular foods or micronutrients. The final nutrient database comprised energy, protein, total fat, total carbohydrate, fibre, vitamin A (retinol equivalents), vitamin C, vitamin B12, folate, thiamine, niacin, riboflavin, zinc, iron and calcium.

Before aggregating back to the commodity groups, weightings were applied to account for the quantity of each food item typically consumed. In the absence of household food consumption data, we used household food expenditure data from a 2015 Living Conditions and Monitoring Survey (CSO, 2016) to determine the quantity of each food item purchased by households per month. Since the survey only collected monetary values for food purchased, we used mean commodity prices for 2015 (Kabwe et al., 2019). The quantity purchased for each food item was determined as follows:

1 $$Q=e/p$$

where:

$Q:$ is monthly food quantity purchased by household (kg).

$e:$ is monthly food expenditure per item (ZMW).

$p:$ is mean commodity price (ZMW) per kg.

The amount spent (equivalent to quantity purchased) on each food item was assumed to be associated with household consumption. Therefore, weighting factors for individual food items were determined as illustrated below.

2 $${W}_{f}=\frac{{Q}_{x}}{\sum x}$$

where:

${W}_{f}:$ is a weighting factor.

${Q}_{x}:$ is the quantity (kg) of food item x purchased.

$\sum x:$ is the sum of food items (kg) in the food category x.

Wherever information to determine the weighting factors were missing due to unavailable consumption or purchase data, an average nutrient value for specific food categories such as cereals others,^1^ tree nuts others, roots others, vegetables, sugars, fruits, meat, pelagic fish, marine fish, and molluscs was calculated and used for individual foods items in that category.

The weighting was performed to avoid overestimating or underestimating nutrient supply because food items are not eaten in the same quantities or weights (Khoury et al., 2014; Sheehy et al., 2019). For example, taking an average of the food group ‘milk excluding butter’ comprises milk, cheese, and yoghurt with very different weights consumed and nutrient profiles, respectively. A comparison was then made for energy, fat and protein in the FBS dataset and the dataset we created by adding micronutrients to the commodities in the FBS (Kapulu et al., 2022), which identified any discrepancies that were then explored and resolved. Nutrient data were then aggregated back to the food commodity groups in the FBS, using the weighting. This gave a nutrient composition for each food commodity in the FBS.

Our estimated nutrient values for energy, protein, and fat were compared with those reported in the FBS values for 2013 to assess the accuracy of these estimated values. As shown in Online Resource 3, the estimates for total energy were on average 6% higher than FBS values, although the correlation was high for the different commodities in the FBS and the values we calculated (r = 0.99, P < 0.0001). The estimated total protein was, on average, 9% higher than FBS values, with a high correlation between the data sources (r = 0.99, P < 0.0001). By contrast, total fat estimates were on average 4% lower than FBS values but highly correlated (r = 0.99, P < 0.0001).

### Trend analysis and estimation of recommended nutrient intakes

The food supply (kcal/cap/d) and population data (1961 – 2013) were compared with the per capita gross domestic product (GDP) from the World Bank (WorldBank, 2019) as a key indicator of the average level of prosperity in Zambia, and nutrient supply of protein, fat and carbohydrate were expressed as absolute amounts and as a percentage of total energy. Further, contributions to nutrients from different foods were categorised into 13 food groups. Finally, values for the food sources of nutrients were presented as an average of 3 years.

Population-weighted estimated average requirement (EAR) for energy and recommended nutrient intakes (RNIs-requirements of 97.5% of the population) were calculated based on age and gender-specific recommendations. The change in population demographics from 1961 to 2013 was factored in to estimate recommended nutritional requirements (FAO/WHO/UNU, 2001). Population demographics remained relatively stable: age 0 – 14 years increased from 46%—47% between 1961 and 2013; age 15 – 64 years reduced from 52%—50%; and 65 years and above reduced from 3%—2%. For protein, zinc, iron, calcium, vitamin A, vitamin C, vitamin B12, folate, niacin, riboflavin, and thiamine, RNIs taken from recommendations from the Joint FAO/World Health Organisation (WHO) (FAO/WHO, 2004; WHO, 2007) were used. We assumed a low bioavailability, 15% and 5% for zinc and iron requirements, respectively, because the diet is predominately plant-based, and these are less bioavailable than for meat-based diets (FAO/WHO, 2004). Average population-level intake requirements for fibre was based on a 20 g per day recommendation, while requirements for fat and carbohydrate were based on 30% and 55% of total energy, respectively (FAO, 2010; WHO, 2003).

### Mapping political, socio-economic and climatic events

The political, socio-economic and climatic events that have occurred in Zambia between 1961 and 2013 were collated by triangulating information from existing literature, government websites, and databases on a timeline of events (CSO, 2019; Elliott & Perrault, 2006; FAOSTAT, 2013; Good, 1989; Harris et al., 2017; Kydd, 1986; NFNC, 2020; Robinson et al., 2007; Sikamo, 2016; Sitko et al., 2018; WorldBank, 2019). Additionally, local expert knowledge was included to supplement information gaps. Finally, these events were used to observe their co-occurrence with dietary nutrient supply trends, including food and nutrition security (see Online Resource 1).

## Results

### Changes in the supply of nutrients between 1961 to 2013 compared with population-level requirements

#### Supply of energy and macronutrients, including food sources

**Fig. 1:**
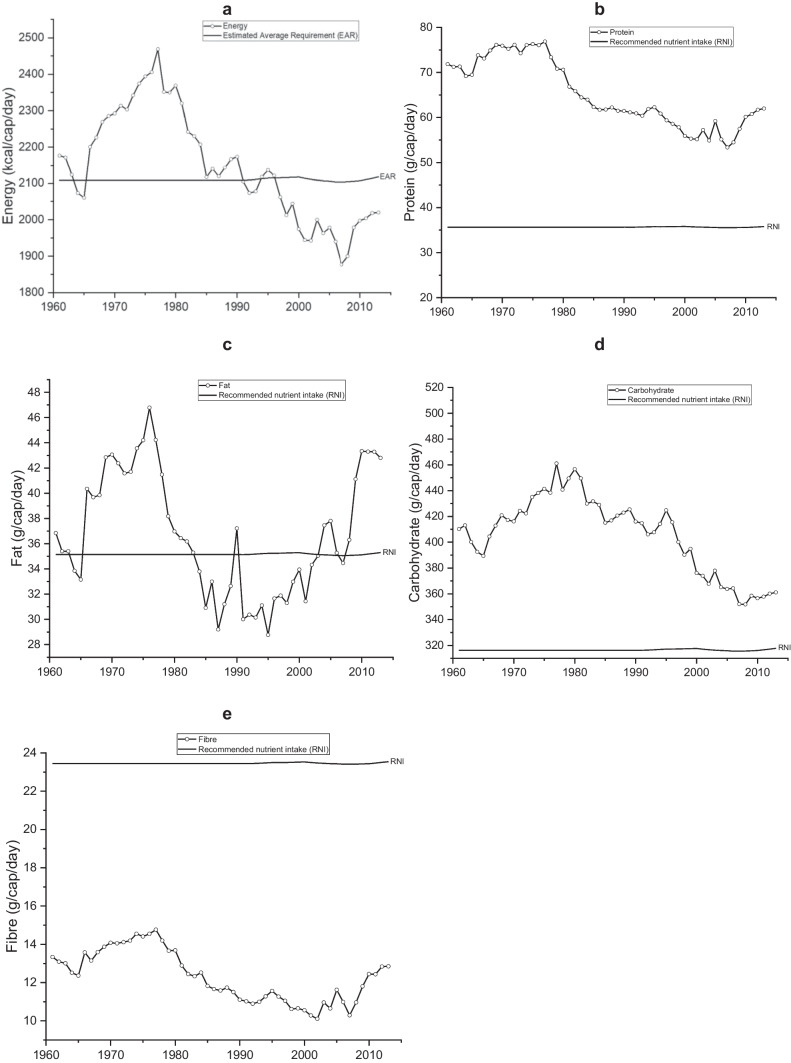
Supply of energy and macronutrients, including estimated average requirements for energy and recommended nutrient intakes for the other macronutrients and micronutrients in Zambia between 1961 and 2013. **a** Per capita energy supply (kcal/d). **b** Per capita protein supply (g/d). **c** Per capita fat supply (g/d). **d** Per capita carbohydrate supply (g/d). **e** Per capita fibre supply (g/d)

Trends in the supply of energy and macronutrients are shown in Fig. 1. Total energy supply increased to a high in the late 1970s, then a steady decline until 2007 to below population requirements. Since 2007 there has been a gradual increase, but the values have remained below EAR. The supply of protein and carbohydrates followed a similar trend to energy, although supply remained higher than requirements. Absolute protein and per cent of energy from protein have been within the average daily population recommendation threshold of 40 g/capita and 11 – 13% of dietary energy, respectively (Roser & Ritchie, 2013; WHO, 2007); supply has been declining since the 1960s. In contrast, total fat followed a similar pattern to energy and ranged between approximately 36 g/cap/d to 43 g/cap/d the entire period. The only period when energy supply from fat was within the 30% of total energy population recommendation is between 1983 and 2003. The supply of fibre follows energy trends, but supply is up to 57% below RNIs in some years throughout the period.

Table 1 shows different food sources contributing to energy and macronutrient supply. Cereals (mainly maize) have dominated the supply of all these nutrients since the 1960s, followed by starchy roots. Over time, however, starchy roots (i.e., cassava), oil crops (i.e., soybean) and sugars have increased contributions to energy, protein and fat supply, although there was a drop in 1988, which later increased. Energy from edible oils and fats also gradually increased, while contributions from cereals, especially maize, declined. Likewise, fruits and vegetable contributions to energy have also gradually decreased with time. Trends show that percentage contributions from animal source foods (ASF) to energy have been low, although it has slightly increased. Protein supply, especially from ASF and cereals, has gradually declined over time. However, a notable increase in contribution from oil crops (mostly soybean) beyond 2008 increased the overall protein supply from 58 g/cap/d to 65 g/cap/d around 2012.

**Table 1:** Food sources for energy and macronutrients, trends, actual quantity per capita and percentage contribution

Energy and macronutrients																		
**Food group**	Food supply			Energy			Protein			Fat			Carbohydrate			Fibre		
	g			Kcal (%)			g (%)			g (%)			g (%)			g (%)		
	**1962**	**1988**	**2012**	**1962**	**1988**	**2012**	**1962**	**1988**	**2012**	**1962**	**1988**	**2012**	**1962**	**1988**	**2012**	**1962**	**1988**	**2012**
Cereals	499.0	464.4	361.1	1576 (73.0)	1458 (68.0)	1153 (57.0)	43.3 (60.6)	40.5 (65.5)	32.0 (52.1)	17.0 (47.4)	15.8 (51.0)	12.0 (27.9)	330.7 (81.1)	306.3 (72.4)	242.2 (67.3)	9.2 (69.6)	8.7 (74.8)	7.0 (55.3)
Starchy roots	139.0	187.3	239.5	171 (7.9)	234 (10.9)	287 (14.2)	1.5 (1.9)	1.9 (3.1)	2.5 (4.0)	0.2 (0.6)	0.3 (1.0)	0.4 (0.9)	40.9 (10.0)	56.2 (10.0)	68.8 (19.1)	0.4 (3.2)	0.5 (3.9)	0.7 (5.6)
Sugars	17.0	46.8	28.4	68 (3.1)	187 (8.7)	114 (5.7)	0.0 (0.0)	0.0 (0.0)	0.0 (0.0)	0.0 (0.0)	0.0 (0.0)	0.0 (0.0)	16.9 (4.1)	46.8 (11.1)	28.6 (8.0)	0.0 (0.0)	0.0 (0.0)	0.0 (0.0)
Pulses	8.1	4.4	5.1	30 (1.4)	16 (0.8)	19 (0.9)	1.6 (2.3)	0.9 (1.4)	1.0 (1.7)	0.5 (1.3)	0.3 (0.8)	0.3 (0.7)	4.8 (1.2)	2.6 (0.6)	3.0 (0.8)	0.3 (2.3)	0.2 (1.4)	0.2 (1.6)
Oil crops	16.1	4.6	31.9	85 (3.9)	24 (1.1)	166 (7.8)	4.2 (5.9)	1.2 (2.0)	9.7 (15.8)	7.1 (19.8)	1.9 (6.1)	10.2 (23.7)	3.0 (0.7)	0.9 (0.2)	7.6 (2.1)	1.4 (10.6)	0.4 (3.5)	3.0 (23.8)
Edible oils & fats	4.4	7.2	14.0	39 (1.8)	64 (3.0)	124 (6.2)	0.0 (0.0)	0.0 (0.0)	0.0 (0.0)	4.3 (12.1)	7.1 (22.9)	13.8 (32.0)	0.0 (0.0)	0.0 (0.0)	0.0 (0.0)	0.0 (0.0)	0.0 (0.0)	0.0 (0.0)
Vegetables	90.2	85.8	73.6	28 (1.3)	27 (1.2)	23 (1.1)	1.6 (2.2)	1.5 (2.5)	1.3 (2.2)	0.2 (0.7)	0.2 (0.8)	0.2 (0.5)	5.3 (1.3)	5.1 (1.2)	4.4 (1.2)	1.2 (9.0)	1.1 (9.8)	1.0 (7.8)
Fruits	34.6	33.0	26.0	16 (0.7)	15 (0.7)	13 (0.6)	0.2 (0.3)	0.2 (0.4)	0.4 (0.3)	0.2 (0.7)	0.2 (0.8)	0.2 (0.4)	3.4 (0.8)	3.2 (0.7)	2.7 (0.7)	0.7 (5.3)	0.7 (6.0)	0.5 (4.0)
Meat	45.4	34.6	39.9	95 (4.4)	73 (3.4)	78 (3.9)	13.8 (19.3)	10.4 (17.4)	10.4 (16.8)	4.1 (11.5)	3.1 (10.2)	3.9 (9.0)	0.8 (0.2)	0.4 (0.1)	0.5 (0.1)	0.0 (0.0)	0.0 (0.0)	0.0 (0.0)
Eggs	2.3	7.2	9.5	3 (0.1)	9 (0.4)	11 (0.6)	0.3 (0.4)	0.8 (1.3)	1.3 (1.7)	0.2 (0.5)	0.6 (1.9)	0.8 (1.8)	0.0 (0.0)	0.0 (0.0)	0.1 (0.0)	0.0 (0.0)	0.0 (0.0)	0.0 (0.0)
Milk & products	35.6	25.9	22.8	30 (1.4)	22 (1.0)	21 (1.0)	1.8 (2.5)	1.3 (2.1)	1.2 (2.0)	1.5 (4.1)	1.1 (3.5)	1.0 (2.4)	1.8 (0.4)	1.3 (0.3)	1.3 (0.3)	0.0 (0.0)	0.0 (0.0)	0.0 (0.0)
Fish	28.9	23.8	18.2	16 (0.8)	14 (0.6)	10 (0.5)	3.2 (4.5)	2.7 (4.3)	2.0 (3.2)	0.4 (1.1)	0.3 (1.0)	0.2 (0.6)	0.0 (0.0)	0.0 (0.0)	0.0 (0.0)	0.0 (0.0)	0.0 (0.0)	0.0 (0.0)
Other foods	0.7	0.6	2.7	1 (0.1)	1 (0.1)	5 (0.2)	0.0 (0.0)	0.0 (0.0)	0.1 (0.2)	0.1 (0.2)	0.0 (0.0)	0.1 (0.2)	0.2 (0.0)	0.2 (0.0)	0.6 (0.2)	0.0 (0.0)	0.1 (0.7)	0.2 (1.8)
Total	921.3	925.6	872.8	2157 (100)	2143 (100)	2041 (100)	71.5 (100)	61.8 (100)	61.5 (100)	35.9 (100)	31.0 (100)	43.1 (100)	407.8 (100)	423.0 (100)	359.6 (100)	13.1 (100)	11.6 (100)	12.7 (100)
Contribution to total energy supply – kcal/g (%)							285.9 (13.3)	247.3 (11.5)	245.9 (12.2)	323.0 (15.0)	279.0 (13.0)	388.2 (19.3)	1631.3 (75.6)	1692.1 (78.9)	1438.6 (71.4)			

#### Supply and food sources of vitamins

**Fig. 2:**
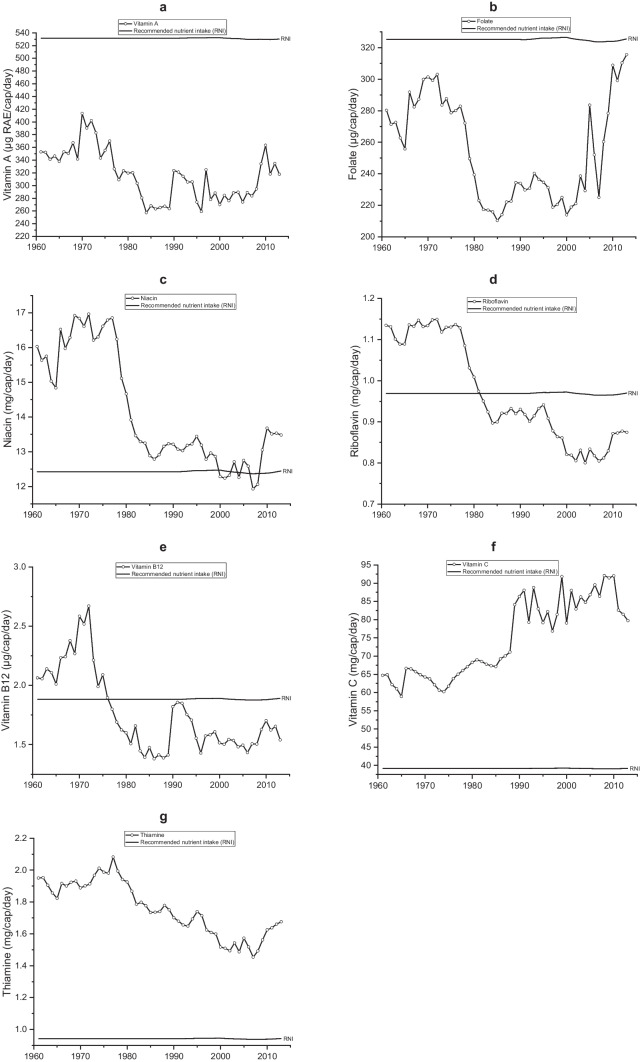
Supply of vitamins, including recommended nutrient intakes in Zambia between 1961 and 2013. **a** Per capita vitamin A supply (µg RAE/d). **b** Per capita folate supply (µg/d). **c** Per capita niacin supply (mg/d). **d** Per capita riboflavin supply (mg/d). **e** Per capita vitamin B12 (µg/d). **f** Per capita vitamin C (mg/d). **g** Per capita thiamine (mg/d)

Figure 2 shows the trends for the supply of vitamins. The supply of folate, niacin, riboflavin, vitamin B12 and thiamine is similar to the energy supply trends. Riboflavin and vitamin B12 fall below requirements in the late 1970s and remains at these levels, but thiamine supply has greatly exceeded the RNIs since 1961. Niacin supply dropped a lot faster than energy in the 1980s, despite being above the RNI. However, the trend for vitamin C shows that it has been gradually increasing, exceeding RNI, from 1961 until 2009, when it levels off. Zambia consistently does not meet its vitamin A and folate requirements. Table 2 shows different food sources and trends for the supply of vitamins. Meat has been the primary source of vitamin B12, although supply reduced gradually. While cereals mostly dominated the source of folate, thiamine, niacin, and riboflavin, supply declined with time as it relatively increased from cassava and soybean. Starchy roots, predominantly cassava, has been a major source (50 – 70%) of vitamin C since the 1960s and the increased supply of cassava over this time explains the increased trends in per capita vitamin C supply. Meat has also accounted for 40—50% of total vitamin A supplied historically, of which nearly 99% came from offal. Contributions to vitamin A from starchy roots have increased slightly over time.

**Table 2:** Food sources for vitamins, trends, actual quantity per capita and percentage contribution

Vitamins												
**Food group**	Vitamin A			Vitamin C			Vitamin B12			Folate		
	µg (%)			mg (%)			µg (%)			µg (%)		
	**1962**	**1988**	**2012**	**1962**	**1988**	**2012**	**1962**	**1988**	**2012**	**1962**	**1988**	**2012**
Cereals	34.6 (9.9)	33.9 (12.8)	25.5 (7.9)	0.0 (0.0)	0.0 (0.0)	0.0 (0.0)	0.0 (0.0)	0.0 (0.0)	0.0 (0.0)	114.1 (41.5)	104.9 (46.3)	83.5 (27.1)
Starchy roots	27.9 (8.0)	27.5 (10.3)	47.4 (14.7)	34.4 (53.8)	46.8 (62.3)	57.9 (71.3)	0.0 (0.0)	0.0 (0.0)	0.0 (0.0)	31.7 (11.6)	41.4 (18.3)	53.5 (17.3)
Sugars	0.0 (0.0)	0.0 (0.0)	0.0 (0.0)	0.0 (0.0)	0.0 (0.0)	0.0 (0.0)	0.0 (0.0)	0.0 (0.0)	0.0 (0.0)	0.0 (0.0)	0.0 (0.0)	0.0 (0.0)
Pulses	0.2 (0.0)	0.1 (0.0)	0.1 (0.0)	0.0 (0.0)	0.0 (0.0)	0.0 (0.0)	0.0 (0.0)	0.0 (0.0)	0.0 (0.0)	29.5 (10.7)	15.9 (7.0)	18.5 (6.0)
Oil crops	0.0 (0.0)	0.0 (0.0)	0.3 (0.1)	0.0 (0.0)	0.0 (0.0)	0.0 (0.0)	0.0 (0.0)	0.0 (0.0)	0.0 (0.0)	38.7 (14.1)	11.4 (5.0)	100.8 (32.7)
Edible oils & fats	16.6 (4.8)	5.9 (2.2)	17.3 (5.4)	0.0 (0.0)	0.0 (0.0)	0.0 (0.0)	0.0 (0.0)	0.0 (0.0)	0.0 (0.0)	0.0 (0.0)	0.0 (0.0)	0.0 (0.0)
Vegetables	59.4 (17.0)	56.3 (21.2)	48.9 (15.1)	17.4 (27.2)	16.4 (21.8)	13.9 (17.1)	0.0 (0.0)	0.0 (0.0)	0.0 (0.0)	29.3 (10.7)	27.9 (12.3)	24.3 (7.9)
Fruits	13.6 (3.9)	13.6 (5.1)	9.6 (3.0)	11.3 (17.6)	11.3 (15.0)	8.6 (10.6)	0.0 (0.0)	0.0 (0.0)	0.0 (0.0)	4.5 (1.6)	4.4 (1.9)	3.5 (1.1)
Meat	183.1 (52.5)	110.3 (41.5)	153.7 (47.5)	0.4 (0.7)	0.3 (0.3)	0.4 (0.4)	1.5 (70.4)	0.9 (62.4)	1.2 (71.8)	16.9 (6.2)	10.5 (4.6)	14.4 (4.7)
Eggs	3.3 (0.9)	10.2 (3.8)	13.1 (4.1)	0.0 (0.0)	0.0 (0.0)	0.0 (0.0)	0.02 (0.9)	0.06 (4.1)	0.07 (4.6)	1.0 (0.3)	3.0 (1.3)	3.9 (1.3)
Milk & products	7.5 (2.1)	5.4 (2.0)	5.2 (1.6)	0.5 (0.7)	0.34 (0.5)	0.3 (0.4)	0.2 (9.4)	0.1 (10.1)	0.1 (8.5)	5.6 (2.0)	4.0 (1.8)	3.9 (1.3)
Fish	2.8 (0.8)	2.3 (0.9)	1.7 (0.5)	0.0 (0.0)	0.0 (0.0)	0.0 (0.0)	0.4 (19.3)	0.3 (23.3)	0.2 (14.9)	3.5 (1.3)	2.8 (1.3)	1.9 (0.6)
Other foods	0.0 (0.0)	0.1 (0.1)	0.7 (0.2)	0.0 (0.0)	0.04 (0.1)	0.1 (0.1)	0.0 (0.0)	0.0 (0.0)	0.0 (0.0)	0.0 (0.0)	0.1 (0.0)	0.2 (0.1)
Total	348.9 (100)	265.6 (100)	323.6 (100)	63.9 (100)	75.1 (100)	81.3 (100)	2.1 (100)	1.4 (100)	1.6 (100)	274.8 (100)	226.4 (100)	308.4 (100)
**Food group**	Thiamine			Niacin			Riboflavin					
	mg (%)			mg (%)			mg (%)					
	**1962**	**1988**	**2012**	**1962**	**1988**	**2012**	**1962**	**1988**	**2012**			
Cereals	1.5 (78.2)	1.4 (80.4)	1.1 (66.4)	9.2 (58.1)	8.5 (64.5)	6.7 (49.6)	0.7 (60.5)	0.6 (59.5)	0.4 (46.7)			
Starchy roots	0.1 (4.4)	0.11 (6.5)	0.14 (8.7)	0.8 (5.3)	1.1 (8.5)	1.4 (10.4)	0.04 (3.3)	0.05 (5.3)	0.06 (7.1)			
Sugars	0.003 (0.2)	0.001 (0.5)	0.006 (0.3)	0.0 (0.0)	0.0 (0.0)	0.0 (0.0)	0.0 (0.0)	0.0 (0.0)	0.0 (0.0)			
Pulses	0.03 (1.6)	0.02 (0.9)	0.02 (1.2)	0.2 (1.0)	0.1 (0.6)	0.1 (0.7)	0.01 (0.9)	0.005 (0.6)	0.006 (0.7)			
Oil crops	0.1 (5.4)	0.03 (1.7)	0.23 (13.6)	2.0 (12.8)	0.5 (3.6)	2.2 (16.3)	0.03 (3.1)	0.01 (1.1)	0.08 (9.2)			
Edible oils & fats	0.0 (0.0)	0.0 (0.0)	0.0 (0.0)	0.0 (0.0)	0.0 (0.0)	0.0 (0.0)	0.0 (0.0)	0.0 (0.0)	0.0 (0.0)			
Vegetables	0.1 (5.5)	0.1 (5.4)	0.07 (4.1)	0.5 (2.9)	0.4 (3.2)	0.4 (2.7)	0.1 (9.2)	0.1 (10.7)	0.09 (9.9)			
Fruits	0.01 (0.5)	0.01 (0.6)	0.009 (0.5)	0.2 (1.0)	0.1 (0.9)	0.2 (1.3)	0.02 (1.7)	0.02 (1.8)	0.01 (1.5)			
Meat	0.1 (2.8)	0.05 (2.7)	0.06 (3.7)	2.3 (14.8)	1.9 (14.4)	2.1 (15.5)	0.2 (13.8)	0.1 (11.9)	0.1 (14.3)			
Eggs	0.0 (0.0)	0.003 (0.)	0.003 (0.2)	0.0 (0.0)	0.0 (0.0)	0.01 (0.1)	0.01 (0.8)	0.03 (3.2)	0.04 (4.3)			
Milk & products	0.02 (0.9)	0.012 (0.7)	0.012 (0.7)	0.04 (0.2)	0.03 (0.2)	0.03 (0.2)	0.06 (5.7)	0.05 (5.0)	0.05 (5.1)			
Fish	0.001 (0.4)	0.006 (0.3)	0.005 (0.3)	0.6 (3.9)	0.5 (3.9)	0.4 (2.9)	0.01 (0.9)	0.008 (0.9)	0.006 (0.7)			
Other foods	0.0 (0.0)	0.0 (0.0)	0.001 (0.1)	0.0 (0.0)	0.01 (0.1)	0.03 (0.2)	0.0 (0.0)	0.0 (0.0)	0.002 (0.3)			
Total	1.9 (100)	1.8 (100)	1.7 (100)	15.8 (100)	13.1 (100)	13.5 (100)	1.1 (100)	0.9 (100)	0.9 (100)			

#### Supply and food sources of minerals

**Fig. 3:**
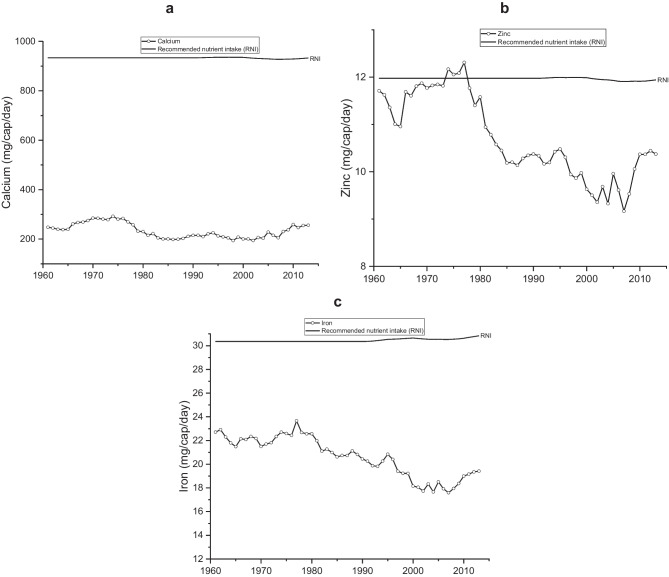
Supply of minerals, including recommended nutrient intakes in Zambia between 1961 and 2013. **a** Per capita calcium (mg/d). **b** Per capita zinc supply (mg/d). **c** Per capita iron supply (mg/d)

The supply of calcium, zinc and iron are shown in Fig. 3. Zinc and iron follow the energy trends, although levels have been below RNIs since the 1960s and have gradually declined until 2007. The trend for calcium is somewhat different from energy, and very little change has occurred to supply over time but has consistently been below the RNI.

Table 3 shows the supply of zinc, iron and calcium from different foods sources (1961 to 2013). Maize has dominated zinc and iron supply, with a small contribution from animal-based products (< 15%). Soybean and cassava have replaced maize, millet, milk and vegetables in the supply of calcium. Soybean, in particular, increased contributions from oil crops towards zinc, iron and calcium, especially after 2005. While a notable dietary shift has occurred, that is, reductions in cereal, meat, vegetable and milk contributions and increases from cassava and soybean (recall Table 1), these changes have had little effect on nutrient supply profiles. The foods have mostly balanced each other out while still not meeting the population's needs.

**Table 3:** Food sources for minerals, trends, actual quantity per capita and percentage contribution

Minerals									
**Food group**	Zinc			Iron			Calcium		
	mg (%)			mg (%)			mg (%)		
	**1962**	**1988**	**2012**	**1962**	**1988**	**2012**	**1962**	**1988**	**2012**
Cereals	7.8 (67.1)	7.2 (70.0)	5.7 (54.4)	17.3 (76.6)	16.1 (76.9)	12.4 (64.1)	82.6 (33.8)	56.3 (27.5)	44.8 (17.7
Starchy roots	0.8 (7.1)	1.1 (11.1)	1.4 (13.3)	1.2 (5.2)	1.6 (7.6)	2.0 (10.2)	33.7 (13.8)	43.9 (21.5)	56.8 (22.5)
Sugars	0.0 (0.0)	0.0 (0.0)	0.0 (0.0)	0.02 (0.1)	0.05 (0.2)	0.1 (0.3)	0.4 (0.2)	0.5 (0.3)	1.5 (0.6)
Pulses	0.3 (2.4)	0.2 (1.4)	0.2 (1.7)	0.3 (1.2)	0.1 (0.7)	0.2 (0.9)	5.3 (2.2)	2.8 (1.4)	3.4 (1.3)
Oilcrops	0.6 (4.9)	0.2 (1.9)	1.5 (14.6)	0.5 (2.3)	0.2 (1.0)	2.0 (10.2)	12.3 (5.0)	4.8 (2.3)	53.5 (21.2)
Edible oils & fats	0.0 (0.0)	0.0 (0.0)	0.0 (0.0)	0.0 (0.0)	0.0 (0.0)	0.0 (0.0)	0.1 (0.0)	0.0 (0.0)	0.0 (0.0)
Vegetables	0.3 (2.3)	0.3 (2.4)	0.2 (2.0)	1.6 (6.9)	1.5 (7.1)	1.3 (6.8)	43.1 (17.6)	41.4 (20.2)	36.8 (14.6)
Fruits	0.1 (0.4)	0.1 (0.5)	0.03 (0.3)	0.1 (0.6)	0.1 (0.7)	0.1 (0.5)	4.9 (2.0)	4.8 (2.3)	3.9 (1.5)
Meat	1.3 (11.5)	0.9 (8.4)	0.1 (9.6)	1.3 (5.8)	0.9 (4.2)	1.0 (5.0)	7.8 (3.2)	6.0 (2.9)	5.9 (2.3)
Eggs	0.03 (0.2)	0.1 (0.8)	0.1 (1.0)	0.04 (0.2)	0.1 (0.5)	0.1 (0.8)	1.1 (0.5)	3.6 (1.7)	4.6 (1.8)
Milk & products	0.4 (3.2)	0.3 (2.6)	0.3 (2.5)	0.02 (0.1)	0.01 (0.1)	0.01 (0.1)	47.4 (19.4)	34.5 (16.9)	33.0 (13.1)
Fish	0.1 (0.8)	0.1 (0.7)	0.1 (0.6)	0.2 (1.0)	0.2 (0.9)	0.1 (0.7)	5.3 (0.1)	4.3 (2.1)	3.1 (1.2)
Other foods	0.0 (0.0)	0.0 (0.0)	0.02 (0.2)	0.0 (0.0)	0.02 (0.1)	0.07 (0.4)	0.2 (0.1)	1.6 (0.8)	5.3 (2.1)
Total	11.6 (100)	10.3 (100)	10.4 (100)	22.6 (100)	20.9 (100)	19.3 (100)	244.0 (100)	204.5 (100)	252.6 (100)

### National supply of energy, population growth and GDP between 1961 and 2013

**Fig. 4:**
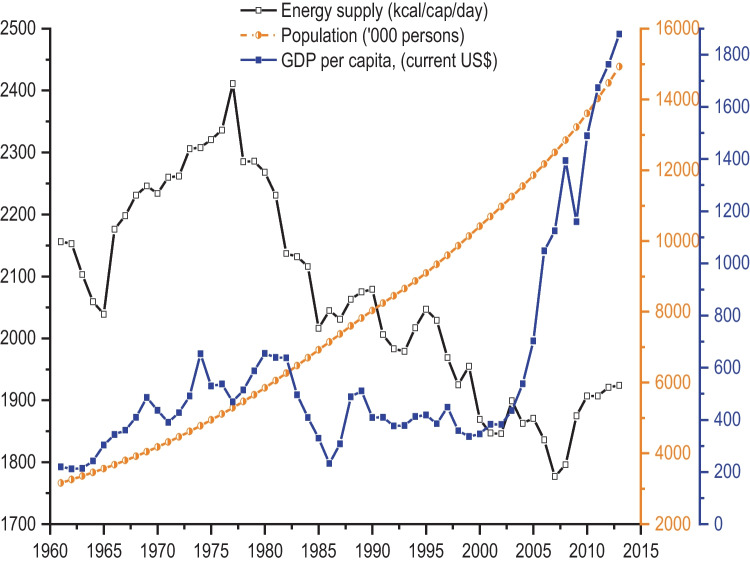
Changes in total population, gross domestic product and energy supply in Zambia between 1961 and 2013. Data sources: (FAOSTAT, 2013; WorldBank, 2019)

The trends in the national energy supply, population and per capita GDP between 1961 and 2013 are shown in Fig. 4. Until the early 2000s, energy supply and GDP followed a similar trend. The daily per capita food supply increased from the mid-1960s to 1977, with the highest supply exceeding 2400 kcal/cap/day in 1977. Since then, energy supplies have declined to about 1800 kcal/cap/day, despite the rapid increase in GDP. Likewise, while between 1961 and 2013 Zambia’s population rapidly increased by almost 300%, the daily per capita energy supply showed gradually declining trends, especially after 1977. This indicates that the energy supply has not kept up with the needs of a rising population.

### Policy, political, socio-economic, and climatic events between 1961 and 2013

A policy analysis was conducted to explore and understand food and nutrition security-related elements (Online Resource 4). There are frameworks for coordinating nutrition interventions implemented by government ministries and agencies in the health, community services, education, agricultural, commerce, trade, and industry sectors. However, emphasis has been placed on the Ministry of Health and National Food and Nutrition Commission to drive the nutrition agenda in the country. As with recent reports (Harris et al., 2017; Manda et al., 2019), the post-2000s have notably emphasised policies addressing food and nutrition insecurity. Particular policy efforts have been made to boost agriculture production diversification to enhance the availability, accessibility and affordability of nutritious foods. Likewise, the policies and plans that address micronutrient deficiencies and child stunting, such as fortification and supplementation, including promoting and consuming nutrient-dense foods, have been developed but are inadequate to mitigate food and nutrition insecurity (Fanzo et al., 2018; Harris et al., 2017). Some of these challenges relate to implementation challenges and poor coordination between and among related ministries (Manda et al., 2018).

**Fig. 5:**
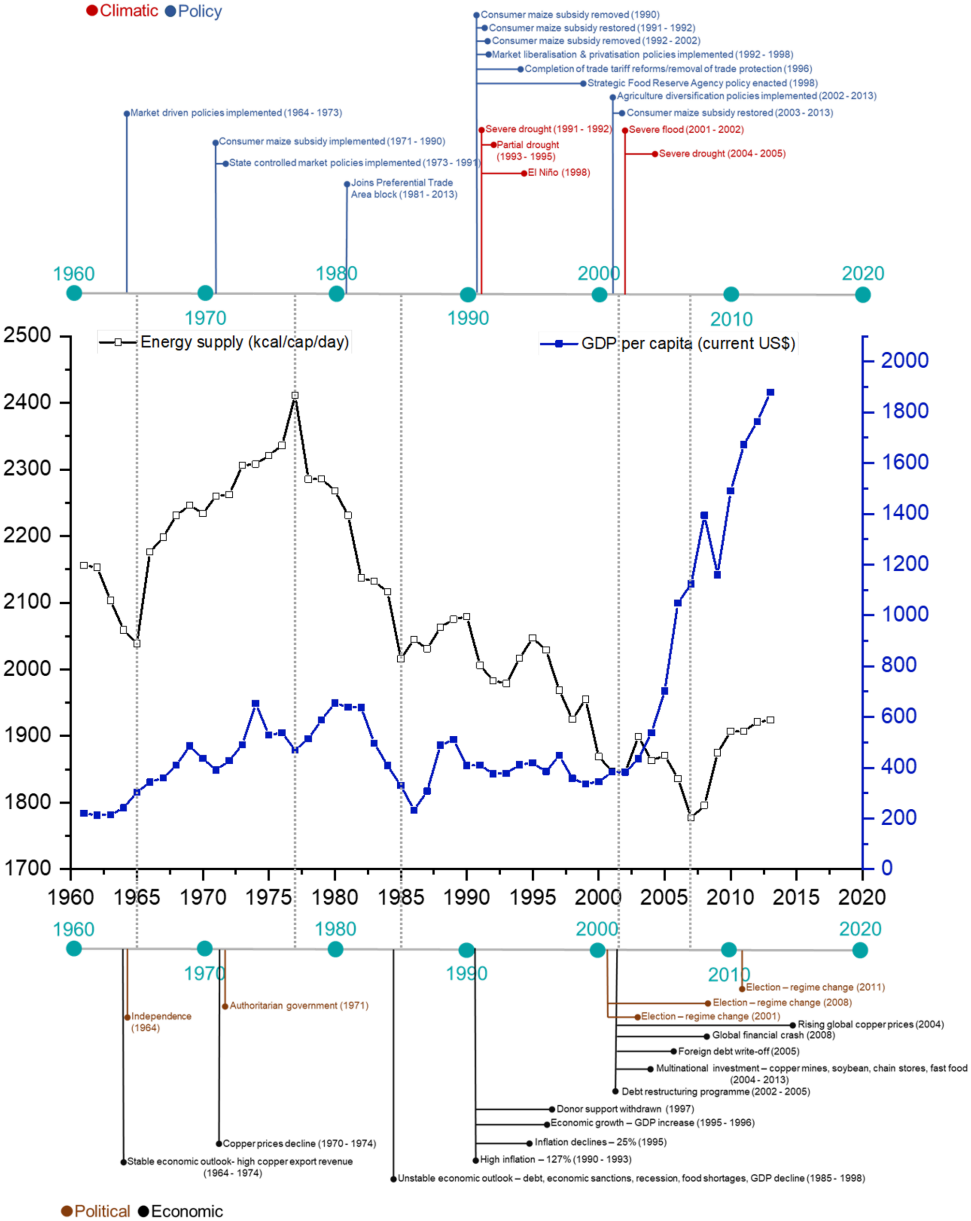
Timeline of key climatic, policy, political and economic events in Zambia between 1961 and 2013, including the trends in energy supply and average per capita GDP (current US$)

Figure 5 shows the political, economic, social, and climate events alongside the changes in energy supply and GDP per capita since the 1960s. Zambia followed authoritarian policies characterised by state-owned enterprises and market regulation from 1975 until 1991. The regime change in 1991 paved the way for implementing neoliberal policies, which saw a more liberalised market economy, privatisation of state-owned enterprises, and lifting of trade sanctions. These policy reforms also initiated the removal of maize subsidies. By the late-1990s, trade reforms were implemented that led to full participation in regional trade blocks such as Preferential Trade Area (later known as Common Market for Eastern and Southern Africa); these helped open regional markets, enhance investments, and stabilise GDP until 2000. Post-2000, increased investment by multinational corporations and foreign debt forgiveness led to increased GDP, yet no change in dietary energy supply was observed. Between 2002 and 2013, various events, including the cancellation of foreign debt, trade tariff reforms, free-market economic policies and agriculture diversification programmes, promoted increased cultivation and processing of more climate-resilient crops such as cassava and soybean. As the economy opened up to internal and external trade, there was a proliferation of supermarkets and fast-food outlets. Multinational investments in soybean after 2004 alongside other economic policies and rising global copper prices (Zambia’s primary export) correspond with a sharp GDP rise and a delayed increase in energy supply from around 2008 (Fig. 5).

Historically, Zambia’s GDP increased from the early-1960s to the mid-1970s, followed by government subsidies for maize, the staple crop (1971–1990). These subsidies were given as agricultural inputs to grow maize and purchase maize mealie meal. During this time, dietary energy supply increased. From the mid-1970s to 1980, the country’s GDP increased and subsequently declined until 1990. This decline corresponds to a fall in global copper prices. In addition, the national debt increased due to an increase in subsidies paid out by the national treasury. During the 1980s, Zambia faced economic sanctions, including restrictions on imports and exports, which led to a recession, losses of foreign revenue and food shortages. These events affected Zambia’s economic stability, and consequently, the country experienced income inequalities, political instability and high poverty levels, especially in urban areas. By the mid to late-1980s, there were food riots due to food shortages and food price hikes.

Notably, the period between 1991 and 2007 was characterised by extreme droughts and floods events, which collectively affected maize production, the predominant energy source (Fig. 5).

## Discussion

This study analysed the FAO FBS for Zambia to assess the population-level adequacy of micronutrients, macronutrients and energy supplies, the sources of nutrients, and changes over time from 1961 to 2013. Throughout the discussion, we will explore the occurrence of socio-economic, political, and climatic events with dietary nutrient supplies during this period concerning food and nutrition security. The observed trends show that most nutrients, except vitamin C, follow similar trends as energy. The supply of energy gradually declined from the late 1970s, falling below the EAR. The supply of micronutrients, including vitamin A, folate, riboflavin, vitamin B12, calcium, zinc, and iron, has been below the RNI since the 1960s. These coincide with specific declines in GDP, economic sanctions, food shortages, high inflation, severe drought and flood events. Maize has historically been central to policies as it dominated diets and contributed 60 – 70% of energy and protein supply. Contributions to nutrients from soybean and cassava increased over time following crop diversification policies during the mid-1990s and since the early-2000s. This improved the supply of calcium, folate and vitamin C. Diets have not changed substantially, although increases in foods such as sugars, fats, and edible oils are emerging, while unrefined cereals, pulses, meat, dairy, vegetables and fruits decreased.

### Trends in energy supply

Daily per capita energy supply is an important indicator of the state of food security (FAO et al., 2019). At a global level, much progress has been made since the 1970s in raising daily per capita energy supply, even among developing countries (von Grebmer et al., 2017). For example, in developing countries, the energy supply increased by nearly 300 kcal/cap/day between the 1960s and late-1990s (Kennedy, 2002). By contrast, but consistent with previous studies, our results showed that for Zambia, energy supply has gradually declined by over 500 kcal/cap/day since 1977, and from the early-1990s, supply was below the EAR (Fig. 1) (Harris et al., 2019; Kapulu et al., 2020; Zhang et al., 2016).

During the period studied, Zambia experienced numerous socio-economic, political, climatic and policy events. In the 1970s and 1980s, the country’s GDP declined as global copper prices fell and economic trade sanctions were imposed. These measures impacted copper and cash-crops (e.g., groundnuts, tobacco and cotton) exports and import of essential commodities, such as wheat, rice, industrial materials, agricultural inputs and fuel, which affected production, processing and distribution of domestically grown food crops, especially maize in the 1980s (Fig. 1). As Zambia lost nearly 30% of its GDP from these economic and copper price shocks, income inequalities, food prices and shortages, and poverty rates increased (Auty, 1991; Elliott & Perrault, 2006), corresponding with a drastic decline in dietary energy supply. Since the mid-1980s, Zambia’s population has also increased rapidly by nearly 120%, alongside increases in urbanisation, which are important causes of high poverty levels, especially in urban areas (Crankshaw & Borel-Saladin, 2018; CSO, 2019) and high prevalence of undernourished people (Nnyepi et al., 2015; Ruel et al., 2008; von Grebmer et al., 2017).

As populations grow, so will food demand if the population energy requirements are to be achieved. This will need either domestic production to increase or a greater reliance on imports. Problems associated with the increasing production of nutrient-dense foods will be more significant with climate change, and trade embargoes and policy shifts affecting the supply of essential food commodities can reduce food availability across the country. Between 1990 and 2013 nearly 50% of the Zambian population was chronically undernourished, with child stunting ranging between 30 – 60%, which could increase further without integrated nutrition, agricultural and trade policies focusing on nutrition security (Fanzo et al., 2018; von Grebmer et al., 2017). In the period after 2000, Zambia implemented several policies such as Vision 2030, National Food and Nutrition Policy (2006), 5^th^ National Development Plan (2006–2010), and 6^th^ National Development Plan (2010–2015) (Online Resource 4). These policies are vital because they provide a framework for economic growth, trade, and investment, explicitly implementing nutrition programmes to address micronutrient and energy gaps. After 2006, energy supply started to increase (Fig. 5), and one possible explanation for this was a rising GDP (> 5% GDP growth) and employment which characterised the mid-2000s due to a resurgence in copper prices and increased investment in the mines. Second, greater investment in maize subsidies following the then Zambian president, Levy Mwanawasa, reintroduced subsidies in 2002 through a Fertiliser Support Programme (FSP) to boost smallscale maize production. This was later preceded by a strong policy focused on improving access by smallscale farmers to input and output markets for maize in the 2004 National Agricultural Policy. Although FSP was later changed to Farmer Input support Programme (FISP), the focus has not changed much from being maize-focused. Consequently, this increased maize production from around 870,000 Mt in 2005 to 2,500,000 Mt in 2013. However, these gains were not adequate to overcome food insecurity simply because the population increased rapidly.

### Micronutrient supply trends

In 1978 Zambia enacted a policy on mandatory fortification of margarine with vitamin A and iron supplementation for pregnant women, while in 1990, vitamin A supplementation was rolled out for children, followed by compulsory household sugar fortification with vitamin A in 1998 (Harris et al., 2017). Zambia’s 1998 mandatory sugar fortification Act was designed to supply nearly 300 vitamin A (RAE) per capita daily (Serlemitsos & Fusco, 2001). However, vitamin A deficiency levels have not reduced much, affecting 54% of children under five years and 13% of adult women due to low intake, inadequate manufacturing compliance monitoring, and degradation (Fiedler et al., 2013; Greene et al., 2017). We did not account for fortification and supplementation in our analysis, and therefore the supply of vitamin A at a population level may be higher than reported here. However, foods like these are not commonly eaten by many in the country; for instance, fortified sugar is only regularly consumed by about 11% of Zambian households (Fiedler et al., 2013). An unintended consequence of using sugar and fats as a vehicle for vitamin A could lead to increased overweight, obesity and diet associated NCDs if consumed in excess. Although previous efforts to fortify maize with vitamin A were piloted in Zambia, this did not succeed due to cost and technological challenges (Fiedler et al., 2013). Thus, supplementation and fortification alone cannot close the nutrient gap and is unlikely to reach the whole population. Therefore, alternatives are increasing production or importing more nutritious foods such as fruits, vegetables, dairy, eggs, nutrient fortified whole grains, pulses and oil crops (i.e., groundnuts and soybean).

We found that the supply of folate, zinc, calcium and iron were consistently lower than RNIs (FAO/WHO, 2004), and vitamin B12 and riboflavin have fallen below the RNI since the early-1980s (Figs. 3–5), consistent with previous findings (Zhang et al., 2016). Household-level surveys that have assessed micronutrient levels report high levels of vitamin A, zinc, iron deficiencies among women (3%, 55%, and 20–31% respectively) and children (35–50%, 22–34% and 35%, respectively) (Schmaelzle et al., 2014; NFNC, 2014; Zambia Statistics Agency et al., 2019). These coincide with the socio-economic, political, climatic, and policy events trade embargoes due to economic sanctions in the 1980s, which meant import restrictions affected the supply of fruits, meat and dairy. However, from 1995, imports for fruits increased when Zambia opened up to trade. Imports play a critical role in supplying nutrients that are not met from in-country production, but this needs to be balanced against exports (Macdiarmid et al., 2018; Schmidhuber et al., 2018). In addition, historically, maize focused policies and subsidies to produce maize to achieve food security (sufficient calories) (Mwanamwenge & Harris, 2017; Gillespie & van den Bold, 2017b) have neglected the need for nutrition security. Failure to diversify domestic food production (e.g., fruit, vegetables, pulses), including population growth, has heavily impacted adequate nutrient supplies.

Zambia has since 1964 demonstrated continued efforts towards establishing a nutrition policy framework to be implemented across institutions and sectors (Online Resource 4). The challenge, however, has been operationalising in a more coordinated manner across various actors (Harris et al., 2017). In addition, changes to the political landscape in the country between 1964 and 2013 (Fig. 5) have yielded inconsistencies in financial commitment towards implementing food and nutrition security-related policies (Harris et al., 2017; Manda et al., 2019). Regime changes in 1991, 2001, 2008, and 2011 have shifted spending priorities to crucial sectors such as health, education and agriculture, which are essential enablers for the successful implementation of policies. However, budgetary allocations have not reflected the importance of mainstreaming nutritional policy (Gillespie & van den Bold, 2017b; Harris et al., 2017). At the same time, evidence shows a slow dietary shift towards energy-dense foods low in fibre, including processed foods, edible oils, and sugars suggesting early nutrition transition stages (Abrahams et al., 2011; Harris et al., 2019; Nnyepi et al., 2015; Popkin et al., 2012). This coincides with rising GDP, economic development, urbanisation, maize-focused agricultural policies such as the FISP, trade liberalisation, and a proliferation of multinational supermarkets and fast-food outlets after 2000 (Chisanga & Zulu-Mbata, 2018; Harris et al., 2019; Nnyepi et al., 2015). Increases in food commodity trade across borders have consequently increased the availability of processed foods through imports.

### Crop diversification in Zambian diets

This study shows that maize has been and remains the dominant source of energy in diets in Zambia (> 50%) and subsequently macronutrients and some micronutrients (e.g., folate, thiamine, niacin, riboflavin iron and zinc), with maize production increasing from below 500,000 Mt to over 3 million Mt in five decades (Chapoto et al., 2016b). However, despite Zambia producing excess maize in some years, such as 2009 to 2013, the country has remained food insecure, which can partly be due to storage-related waste and illegal exports to neighbouring countries along Zambia’s porous borders (Dorosh et al., 2009), even in years with drought and flood events such as 1991, 1995, 2001 and 2004 (Fig. 5). Also, because maize produced countrywide is primarily centralised in Lusaka and a few provincial capitals, accessibility by the poor is difficult, especially during lean months in-between harvests. Furthermore, maize production in Zambia is predominantly rainfed (Mwanamwenge, 2017). Therefore, it is susceptible to the co-occurrence of extreme weather events such as droughts and floods, as seen in 1991/2, 1995, 2001 and 2004/5 reduced maize supply due to low production and yields. These pose severe consequences for food and nutrition security. Thus, diversifying diets is necessary to mitigate against adverse effects caused by external shocks to food supply on nutrient availability.

Cassava production has been promoted through government policy, such as Agriculture Sector Investment Plan (1995–2001) and National Agricultural Policy (2004–2015) and increased donor support since the 1990s as an alternative energy source to maize from a crop/food diversification perspective and to mitigate against extreme weather events like droughts (Chitundu et al., 2006, 2009; Haggblade et al., 2012). According to Jayne et al. (2007) cassava production has doubled and has been considered a staple food for nearly 30% of the Zambian population since the early 1990s. Our findings show that by 2012 cassava’s contribution to national caloric supply had increased from 7% to roughly 15%, consistent with the findings of Chitundu et al. (2009). Our analysis reveals that most vitamin C came from starchy roots, notably cassava, gradually increasing between 1991 and 2010. For instance, after the 1991–2 policy reforms that saw a withdrawal of government maize input and consumer subsidies, maize production declined. Cassava was seen as an alternative low input energy source for many subsistence households, especially in northern and western Zambia (Chitundu et al., 2009; Jayne et al., 2007).

In addition, soybean, a nutrient-dense crop with multiple utilisation options, is rapidly becoming a dominant contributor to the food supply for human consumption and livestock feeds in Zambia. Our analysis revealed a notable increase in oil crops’ (mostly soybean) contribution to energy, protein, carbohydrate, fibre, zinc, iron, calcium, folate, thiamine, niacin, and riboflavin; especially since 2008. Several related events coincided with the exponential growth in soybean contributions to energy nutrients in Zambia. Likewise, post-2000, agriculture diversification, nutrition and trade policies (Fig. 5 and Online Resource 4) were enacted by the government that supported crop diversification away from maize dependence, facilitated land access for foreign agribusinesses, enhanced trade, and paved the way for increased investment in soybean processing, marketing, and utilisation (Chapoto et al., 2016a; Sitko et al., 2018). Since the mid-2000s, the Zambian economy opened up to multinational corporations that invested in chain stores and food processing, including edible oil refining and soybean food processing (Sitko et al., 2018).

Soybean could, therefore, directly contribute to improving Zambia’s food and nutrition security through the supply of these nutrients provided it is consumed domestically. Indirectly, soybean could also contribute to nutrition through livestock products, provided these foods are accessible and affordable even for the poorest. Furthermore, increasing demand and market availability for soybean has fuelled its cultivation among smallholder rural households. Thus, soybean provides an opportunity to diversify away from maize and increase nutrient intake in the Zambian diet. However, to avoid reducing crop diversification, efforts should be placed on promoting soybean growing alongside other crops and not as a monoculture.

### Lesson learned and areas for action

This study has shown how socio-economic, policy, political and climatic events coincide with the food and nutrition security trends. Although the findings are specific to Zambia, they apply to other SSA countries. From a policy perspective, Zambia already has an existing nutrition policy framework (Online Resource 4), but what has lacked is consistent political and economic support towards implementation (Harris et al., 2017). However, it will require integration of nutrition, agricultural and climate change policies. Increasing budgetary and operational support to nutrition from the existing less than 1% of the national budget to even 5% (Harris et al., 2017), followed by total disbursement, could strengthen collaboration and coordination across sectors, including nutrition. This will ensure that existing policies, such as efforts to mainstream nutrition-sensitive agriculture into the country’s agricultural implementation plans, receive adequate budgetary support from the government. A key lesson from this study is that Zambia has historically experienced dietary nutrient supply gaps, especially micronutrients, despite the policy efforts.

There is a need to promote diversified diets rather than just increasing caloric intake through upscaling supply of affordable energy-rich but nutrient-dense foods such as fruit, vegetable, dairy, meat, soybean, groundnuts, nutrient fortified whole grain products, including those that cannot be sourced domestically via imports. In addition, governments can offer tax incentives on domestically produced and imported energy-rich and nutrient-dense foods while imposing higher taxes on less nutritious foods. This would perhaps increase the supply of affordable, nutritious foods. All these require deliberate policy, political and economic support to implement.

Further, our results demonstrate how diversifying agriculture production, e.g., from maize to cassava and soybean, can enhance the supply of some nutrients. However, more is needed to realign budgetary priorities in agriculture, from the production of staples to achieve energy security to nutrition-sensitive agriculture interventions. Lastly, as demonstrated here regarding a co-occurrence of extreme weather events with a decline in nutrient supplies, there is a need to strengthen support towards enhancing production, local-level processing and utilisation of low-input or climate-resilient commodities such as cassava, sorghum, cowpea, common beans, and soybean.

These enablers, taken together, will help facilitate the supply of affordable energy-rich and nutrient-dense foods even among the poor and mitigate caloric and micronutrient deficiencies. However, in the long term, to sustain the supply of affordable sources of key micronutrients and energy in the food system, a stable political and policy environment seems important. Previously, regime change has been disruptive to the implementation of policies and programmes in Zambia.

### Limitations of the study

There are several limitations linked to this study. FBS give national average quantities of energy and macronutrients supply available for consumption, not actual consumption. However, to partly compensate, we adjusted for household food waste. FBS do not consider how nutrients are distributed across the country and that the supply of nutrients might differ between and within households in rural and urban settings. However, country-level data on food consumption at the individual level in Zambia is absent, and surveys are expensive and time-consuming to carry out. Studies that have previously collected consumption or food expenditure data were specific to certain country regions (Mason & Jayne, 2009; CSO, 2016; NFNC, 2014; Hichaambwa et al., 2009; CUTS & WFP, 2018; Kaliwile et al., 2019). Although FBS do not account for actual consumption and how nutrients are distributed within households, the purpose of the study was to provide a national-level perspective of adequacy trends for the supply of micronutrients, energy and other macronutrients. This is essential for tracking dietary trends in the absence of food consumption data and developing appropriate policies and interventions.

## Conclusion

The overall objective of this research was to explore the evolution of energy and nutrient supply in Zambia between 1961 and 2013 in the context of policy, political, social, economic, and climatic changes. First, the study assessed changes in the supply of energy, macronutrients and micronutrients, and the findings show that Zambia was nutrition insecure for key macro- and micronutrients from 1961 to 2013 and energy deficient since the late-1980s. Second, key social, policy, political, economic and climatic events in Zambia to the supply of dietary nutrients, food and nutrition security across the same period were mapped. The study reveals that the supply of nutrients and composition of the diet coincides with key social, economic, policy, political and climatic changes. For example, droughts, floods, population growth, political instability, inconsistent food and nutrition policy implementation and economic shocks created nutrient supply gaps, reflected nationally. Third, the study sought to identify lessons based on historical trends to inform the design of appropriate nutrition policy in Zambia. Consistent with global nutrition transition trends since the early 1990s, Zambia’s food system is influenced by a policy drive towards achieving caloric sufficiency that promotes energy-dense foods with little impact on food security. Likewise, the emergence of supermarkets and improved food processing capacity, alongside increasing per capita GDP and a rising population, has increased the consumption of sugars, edible oils, and fats in the diet. This nutrition transition is increasing the prevalence of obesity and NCDs. More urgently, policy interventions focusing on enhancing nutrition security through promoting more diverse and affordable energy and nutrient-dense foods—such as such as fruit, vegetable, dairy, meat, edible insects, fish, soybean, groundnuts, nutrient fortified whole grain products are required. Going forward, this research recommends that where domestically production does not meet dietary nutrient requirements, increasing the supply of affordable but nutritious foods could be achieved through imports, but this may require tax incentive policies. Lastly, this study underpins advocacy for greater commitment to implementing existing nutrition policies, alongside consistent political and economic support and the integration of nutrition, agricultural and climate change policy to maximise impact on nutrition security. This is fundamental for Zambia to achieve the second UN Sustainable Development Goal to end hunger and all forms of malnutrition by 2030.

## Supplementary Information

Below is the link to the electronic supplementary material.

Supplementary file1 Online Resource 1 A summary of steps taken to estimate energy, micronutrient and macronutrient supplies in Zambia using FAO food balance sheets (1961–2013), including triangulation of co-occurrence of trends with socio-economic, policy, political and climatic events from 1961 to 2013. (PDF 95 KB)

Supplementary file2 Online Resource 2 A list of 264 food items included in the nutrient database and their edible portion conversion factor. (PDF 261 KB)

Supplementary file3 Online Resource 3 Correlations between the FAO food balance sheets (2013) and our estimated values. a per capita energy supply (kcal/d) relationship. b Per capita protein supply (g/d) relationship. c Per capita fat supply (g/d) relationship. (PDF 65 KB)

Supplementary file4 Online Resource 4 Relevant food and nutrition security policies and plans in Zambia (1991 – 2021). (PDF 171 KB)

## Data Availability

The data that support the findings of this study are openly available in the White Rose Repository at Kapulu et al. (2022): Estimated energy, macronutrient and micronutrient supply for Zambia 1961 to 2013 – raw data. [Dataset] https://doi.org/10.5518/1111

## Footnotes

1 Food items that may not otherwise be specifically identified or classified separately in the food balance sheets because of their minor importance internationally.
