# Supplementary figures and images for: Evolution of energy and nutrient supply in Zambia (1961–2013) in the context of policy, political, social, economic, and climatic changes
Source: Food Secur. 2022 Nov 22;15(2):323–42. doi: 10.1007/s12571-022-01329-1 (PMC10066153; doi:10.1007/s12571-022-01329-1)

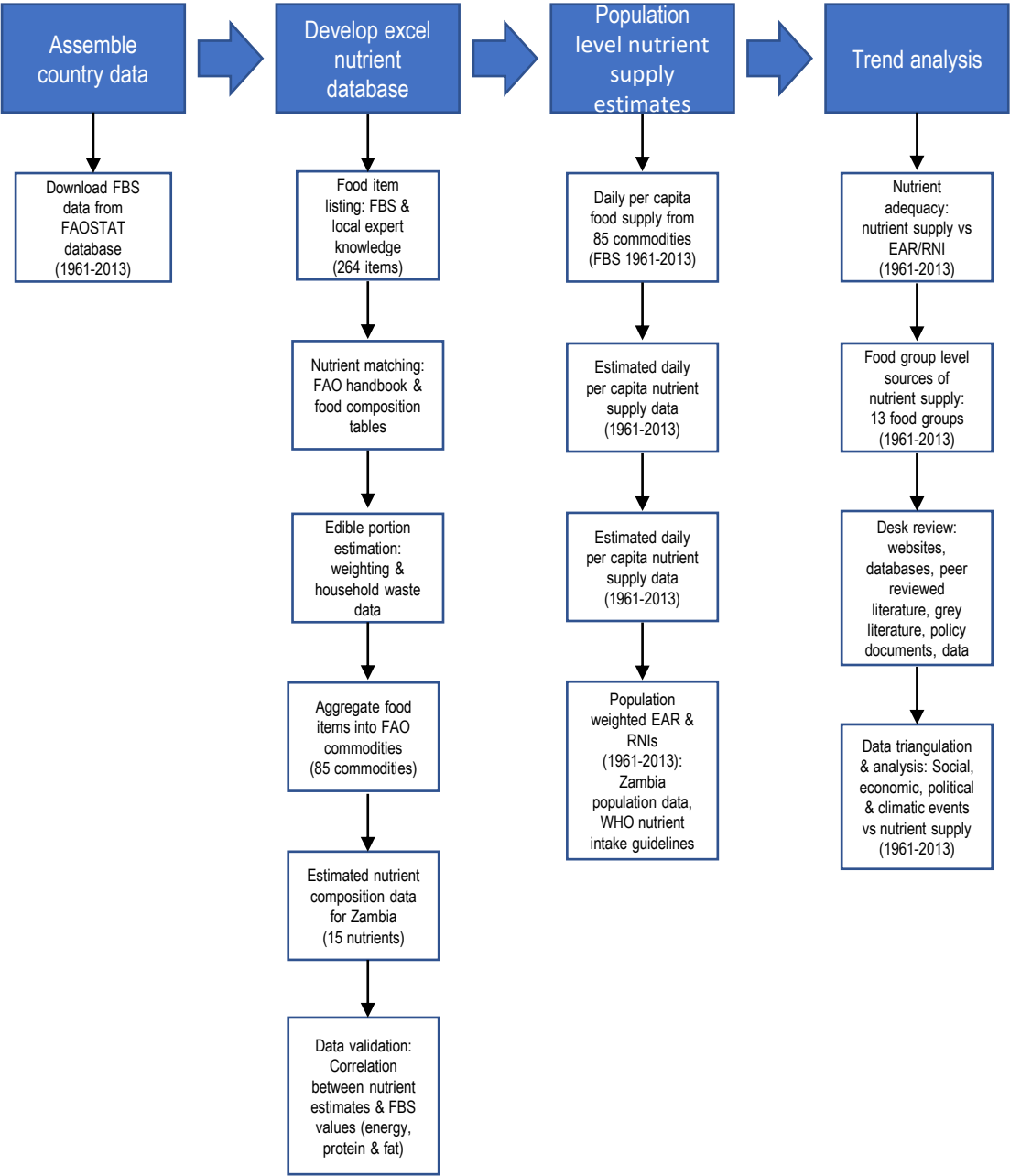

Supplement: Supplementary file 1 — Supplementary file1 Online Resource 1 A summary of steps taken to estimate energy, micronutrient and macronutrient supplies in Zambia using FAO food balance sheets (1961–2013), including triangulation of co-occurrence of trends with socio-economic, policy, political and climatic events from 1961 to 2013. (PDF 95 KB) [file 12571_2022_1329_MOESM1_ESM.pdf]

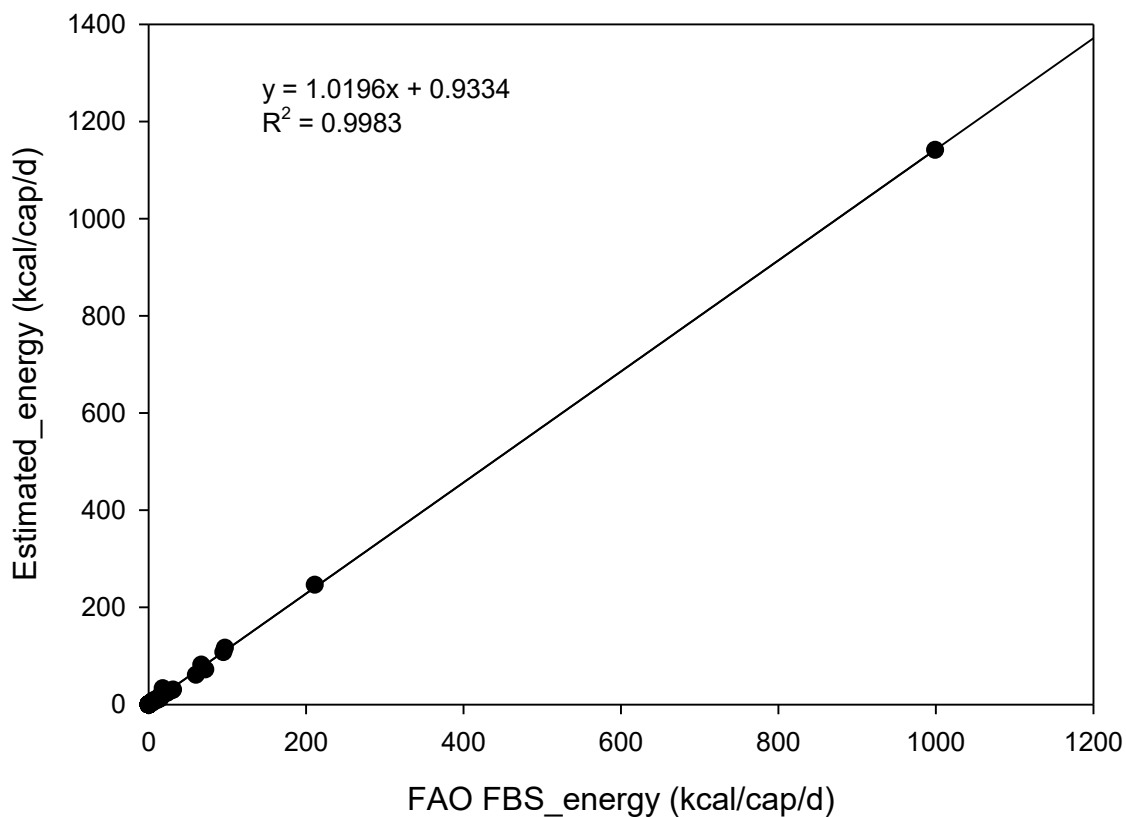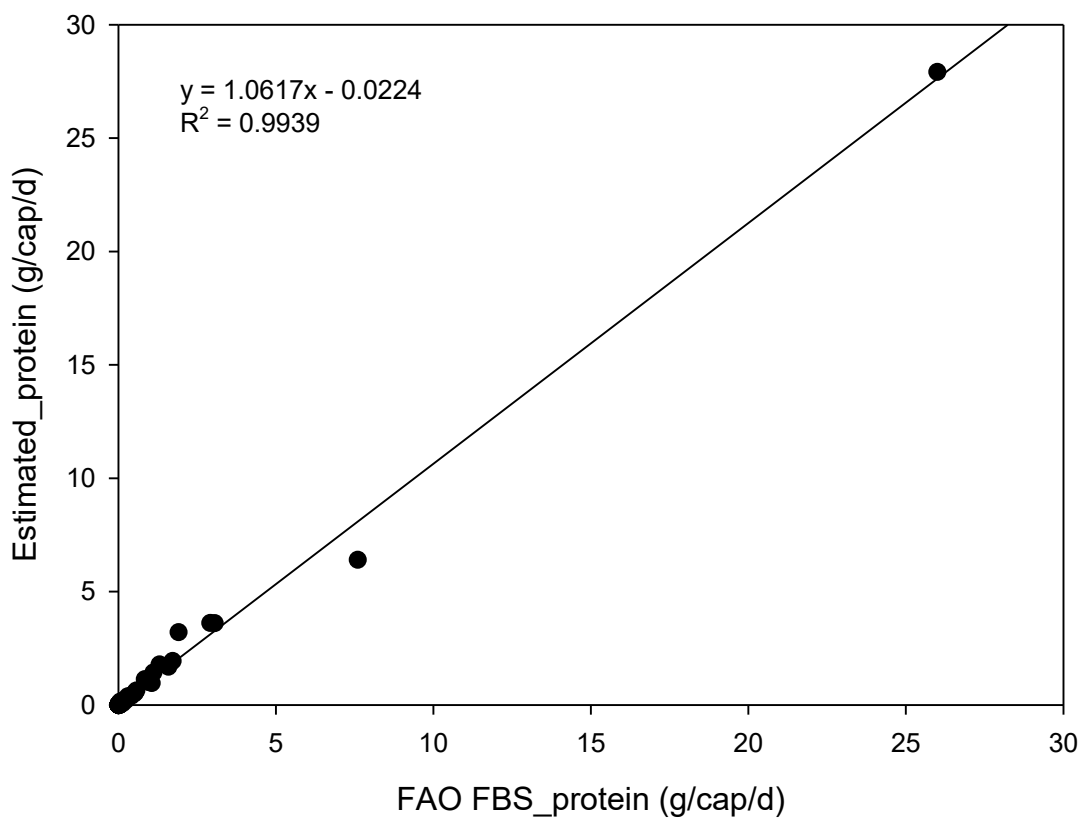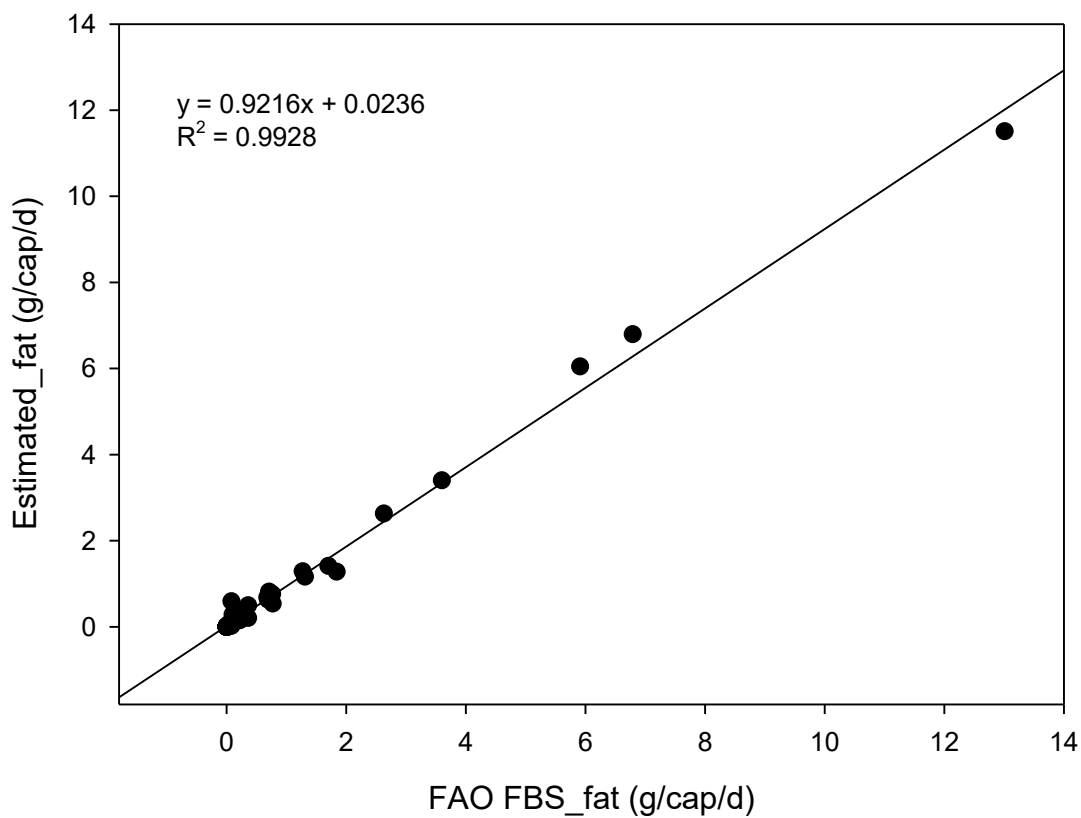

Supplement: Supplementary file 3 — Supplementary file3 Online Resource 3 Correlations between the FAO food balance sheets (2013) and our estimated values. a per capita energy supply (kcal/d) relationship. b Per capita protein supply (g/d) relationship. c Per capita fat supply (g/d) relationship. (PDF 65 KB) [file 12571_2022_1329_MOESM3_ESM.pdf]
