# Supplementary material for: Evolution of energy and nutrient supply in Zambia (1961–2013) in the context of policy, political, social, economic, and climatic changes
Source: Food Secur. 2022 Nov 22;15(2):323–42. doi: 10.1007/s12571-022-01329-1 (PMC10066153; doi:10.1007/s12571-022-01329-1)
Supplement: Supplementary file 2 — Supplementary file2 Online Resource 2 A list of 264 food items included in the nutrient database and their edible portion conversion factor. (PDF 261 KB) [file 12571_2022_1329_MOESM2_ESM.pdf]

A list of 264 food items included in the nutrient database and their edible portion conversion factor

Food item	Edible portion factor (%)
Cereals and products	
1. Wheat flour, 70% extraction	100
2. Wheat flour, 80% extraction	100
3. Wheat flour, whole grain	100
4. Macaroni, dry	100
5. Finger millet flour, 70% extraction	100
6. Finger millet flour, whole grain	100
7. Bulrush millet flour, 80% extraction	100
8. Bulrush millet, whole grain	100
9. Sorghum flour, 66% extraction	100
10. Sorghum flour, whole grain	100
11. White rice	100
12. Brown rice	100
13. Oats	100
14. Oats, rolled	100
15. Barley	100
16. Corn flakes	100
17. Cereal ready-to-eat cereal, lower sugar	100
18. Cereals ready-to-eat, WEETABIX whole grain cereal	100
19. Crispy Brown Rice Cereal	100
20. Muesli, dried fruit and nuts	100
21. Maize flour white whole	100
Tree nuts and products	
1. Almonds, shelled	100
2. Cashew nuts, shelled	100
3. Walnuts, shelled	45
4. Coconuts, mature kernel	52
5. Pistachios	50
Root, tubers and products	
1. Cassava tuber, fresh	100
2. Potatoes	100
3. Sweet potato, orange	84
4. Sweet potato, white	84
5. Sweet potato, yellow	84
6. Orchid roots, *Satira Siva*	84
7. *Dioscorea Hirtiflora*, roots	84
8. Coco yam	84
9. Kasona yam	84
10. Aerial yam	84
11. Njamva yam	84
12. West African yam	84
13. Wild bitter yam	84
14. Yam Dioscorea Species	84
Pulse and products	
1. Butter bean, large seeded	100
2. Dark red beans	100
3. White beans	100
4. Navy purple seed beans	100
5. Haricot beans	100
6. Beans, yellow	100
7. Cowpea	100
8. Pigeon peas	100

9. Green peas, dry	100
10. Lentils	100
11. Bambara beans	100
12. Chick peas	100
Oil crops and products	
1. Soybeans	100
2. Groundnuts	100
3. Palm kernel	100
4. Sesame	100
5. Sunflower	100
6. Coconut (copra)	100
7. Castor beans	100
8. Olives	100
9. Mustard seed	100
10. Soybean oil	100
11. Groundnut oil	100
12. Sunflower seed oil	100
13. Cotton seed oil	100
14. Palm kernel oil, refined	100
15. Palm kernel oil, red	100
16. Coconut oil	100
17. Sesame seed oil	100
18. Margarine, reg, 80% fat	100
19. Rape seed oil	100
20. Mustard seed oil	100
21. Maize germ oil	100
Vegetables and products	
1. Cabbages	88
2. Chinese cabbage	88
3. Rape	88
4. Asparagus	53
5. Lettuce and chicory	70
6. Spinach	72
7. Wild spinach	72
8. Tomatoes raw fruit	91
9. Tomatoes, peeled	100
10. Broccoli	61
11. Cauliflower	61
12. Pumpkin squash, yellow flesh	70
13. Pumpkin squash, pale flesh	70
14. Garlic	87
15. Gourds	70
16. Cucumber	81
17. Eggplants	80
18. Sweet peppers, red	83
19. Sweet peppers, green	83
20. Spring onion	91
21. Onion, large	91
22. Leeks	44
23. Green beans	83
24. String beans	88
25. Carrots	89
26. Turnips	89
27. Green maize, white	36
28. Green maize, yellow	36

29. Wild mushrooms, red-yellow	97
30. Wild mushrooms, brown large	97
31. Mushrooms	97
32. Wild mushrooms, large white	97
33. Dried wild wood mushrooms, brown	100
34. Dried wild mushrooms, red-yellow	100
35. Dried wild mushrooms, brown	100
36. Dried wild mushrooms, large white	100
37. Cassava leaves	85
38. Okra fingers	86
39. Swiss chard	72
40. Baobab leaves	82
41. Amaranth, leaves raw	94
42. Black jack	80
43. Black night shade	80
44. Okra leaves	80
45. Cat wiskers (bush okra)	80
46. Jute (bush okra), raw	80
47. Cowpea leaves	80
48. Moringa	80
49. Pumpkin leaves	41
50. False sesame leaves	100
51. Sweet potato leaves	80
52. Watermelons	52
53. Dry cucurbits	100
54. Dried black jack	100
55. Dried okra fingers	100
56. Dried peppers, red	100
57. Dried pumpkin leaves	100
58. Dried *Zanthoxylum Chalybeum*	100
59. Dried *Hibiscus Meeusei*	100
60. Dried Jute leaves	100
61. Dried bean leaves	100
62. Dried baobab leaves	100
63. Dried cowpea leaves	100
64. Canned tomatoes, packed in juice	100
65. Onions, dry	100
66. Tomato, powder	100
67. Frozen vegetables, mixed	100
Fruits and products	
1. Apples	90
2. Pineapples	51
3. Bananas	64
4. Grape	58
5. Plantains	65
6. Lemons and limes	64
7. Oranges	73
8. Tangerines	73
9. Clementines	73
10. Grapefruit and pomelo	46
11. Dates	90
12. Apricots	80
13. Avocados	74
14. Cherries	70
15. Figs	99

16. Jube jube<br>17. Cranberries<br>18. Blueberries<br>19. Currants<br>20. Raspberries<br>21. Gooseberries<br>22. Mango, deep orange flesh<br>23. Mango, orange flesh<br>24. Mango, pale flesh<br>25. Papayas<br>26. Peaches<br>27. Nectarines<br>28. Pears<br>29. Plums and sloes<br>30. Strawberries<br>31. Guava<br>32. Kiwi<br>33. Melons	93<br>95<br>98<br>98<br>96<br>75<br>71<br>71<br>71<br>62<br>80<br>80<br>77<br>81<br>86<br>78<br>75<br>51
Meat and products<br>1. Corned beef, canned<br>2. Beef, meat, lean boneless, raw<br>3. Beef, meat, lean boneless, raw, 15-20% fat<br>4. Beef, meat, lean boneless, raw, 30% fat<br>5. Beef tripe raw<br>6. Beef ground, raw, 10% fat<br>7. Beef lean<br>8. Beef, mince moderate fat<br>9. Beef, meat, lean, cured, dried<br>10. Chicken dark meat, flesh, skin, raw<br>11. Chicken dark meat, flesh, raw<br>12. Chicken light meat, flesh, raw<br>13. Chicken light meat, flesh, skin, raw<br>14. Game meat, antelope<br>15. Game meat, dried<br>16. Goat meat, raw<br>17. Pork, lean<br>18. Pork meat, boneless, raw, 20% fat<br>19. Pork meat, boneless, raw, 40% fat<br>20. Mutton, lean<br>21. Beef liver, raw<br>22. Beef kidney, raw<br>23. Chicken liver, raw<br>24. Chicken giblets, raw<br>25. Rabbit meat<br>26. Chicken eggs<br>27. Lard	 100<br>98<br>98<br>98<br>98<br>100<br>98<br>100<br>100<br>80<br>80<br>80<br>80<br>52<br>100<br>75<br>80<br>80<br>80<br>75<br>100<br>100<br>100<br>100<br>60<br>88<br>100
Fish and fisheries products<br>1. Tilapia<br>2. “Kapenta”<br>3. African carp<br>4. Cat fish<br>5. Nile perch<br>6. Tigerfish<br>7. Mackerel<br>8. Tuna	 65<br>52<br>54<br>52<br>61<br>54<br>71<br>58

9. Sardine<br>10. Haddock<br>11. Cod<br>12. Bluefish<br>13. White grouper<br>14. Molluscs, other	52<br>67<br>67<br>67<br>67<br>20
Milk and products<br>1. Whole cow milk<br>2. Skimmed whole cow milk, dry<br>3. Skimmed cow milk, 1.5% fat<br>4. Skimmed cow milk, 0.5% fat<br>5. Whole cow milk, dried<br>6. Goat milk<br>7. Cheddar cheese, cow milk<br>8. Gouda cheese, cow milk<br>9. Yoghurt, cow milk<br>10. Whipping cream, 38% fat<br>11. Cream, 13% fat<br>12. Ice cream	 100<br>100<br>100<br>100<br>100<br>100<br>100<br>100<br>100<br>100<br>100<br>100
Beverages Fermented<br>1. "Munkoyo" – fermented<br>Beverages Alcoholic<br>1. "Munkoyo"<br>2. "Chibuku"<br>3. Maize<br>4. Millet<br>5. Sorghum<br>Wine<br>1. Red<br>2. White<br>Beer<br>1. Beer of barley	 100<br><br>100<br>100<br>100<br>100<br>100<br><br>100<br>100<br><br>100
Stimulants<br>1. Coffee green<br>2. Coffee roasted<br>3. Coffee substitutes<br>4. Tea<br>5. Tea mate<br>6. Cocoa butter<br>7. Cocoa, powder, and cake	 100<br>100<br>100<br>100<br>100<br>100<br>100
Sugar and syrups<br>1. Honey<br>2. Sugar<br>3. Sugar cane<br>4. Maple syrup<br>5. Molasses	 100<br>100<br>100<br>100<br>100
Spices<br>1. Black pepper<br>2. Pimento/chilli pepper<br>3. Ginger<br>4. Cinnamon<br>5. Cloves<br>6. Nutmeg<br>7. Spices, nes	 100<br>100<br>100<br>100<br>100<br>100<br>100
