# Supplementary material for: Evolution of energy and nutrient supply in Zambia (1961–2013) in the context of policy, political, social, economic, and climatic changes
Source: Food Secur. 2022 Nov 22;15(2):323–42. doi: 10.1007/s12571-022-01329-1 (PMC10066153; doi:10.1007/s12571-022-01329-1)
Supplement: Supplementary file 4 — Supplementary file4 Online Resource 4 Relevant food and nutrition security policies and plans in Zambia (1991 – 2021). (PDF 171 KB) [file 12571_2022_1329_MOESM4_ESM.pdf]

Relevant food and nutrition security policies and plans in Zambia (1991 – 2021).

Document	Description	Key governmental actor (s)	Year (s)	Strategic focus
Food Reserve Act	Act	Ministry of Agriculture	1995 & 2004	• Strategic food reserves<br>• Stabilise food prices and guarantee food security*
Agriculture Sector Investment Plan	Investment plan	Ministry of Agriculture	1995 - 2001	Improve household food security*
Zambian National Strategy and Plan of Action for the Prevention and Control of Vitamin A Deficiency and Anaemia	Strategic plan	Ministry of Health,<br>National Food and Nutrition Commission,<br>Ministry of Commerce, Trade and Industry	1999-2004	• Micronutrient supplementation and fortification to improve nutrition security*<br>• Micronutrient deficiency prevention and control to improve nutrition security*
National Agricultural Policy	Agricultural policy	Ministry of Agriculture and Livestock	2004-2015	• Diversify agricultural production and utilisation to improve food and nutrition security*<br>• Establish food and nutrition unit (2006) to address nutrition security
Fifth National Development Plan	Investment plan	All sectors	2006-2010	• Invest in sustainable food and nutrition security programmes<br>• Develop national food and nutrition plan*<br>• Establish a functional multi-sectoral nutrition coordination framework*<br>• Create nutrition awareness
Vision 2030	Investment plan	Ministry of Health<br>Ministry of Education,<br>Ministry of Agriculture and Livestock	2006-2030	• Promote healthy diets<br>• Prevent macro and micronutrient deficiencies<br>• Improve agricultural production, processing and trade to address food and nutrition security
National Food and Nutrition Policy	Nutrition policy	Ministry of Health,<br>National Food and Nutrition Commission,<br>Ministry of Agriculture,<br>Ministry of Community Development and Social Services,<br>Ministry of Education	2008	Guideline for developing and implementing nutrition interventions
Sixth National Development Plan	Investment plan	All sectors	2010-2015	• Promote nutritious diets<br>• Promote crop diversification to improve food and nutrition security*<br>• Enhance food processing and utilisation to improve household food and nutrition security<br>• Support nutrition education
National Food and Nutrition Strategic Plan	Plan	Ministry of Health,<br>Ministry of Education,<br>Ministry of Community Development, Mother and Child Health,<br>Ministry of Agriculture and Livestock<br>Ministry of Commerce, Trade and Industry	2011-2015	• Prevent child stunting*<br>• Improve food and nutrition security<br>• Improve nutrition education<br>• Control and prevent diet-related NCDs<br>• Enhance coordination of nutrition interventions*<br>• Promote nutritional messages*
Strategic Plan Non-Communicable Diseases and their Risk Factors	Plan	Ministry of Health	2013-2016	• Guidelines for food processors*<br>• Nutrition labelling<br>• Address NCDs<br>• Promote healthy and nutritious diets*
National Social Protection Policy	Policy	Ministry of Community Development, Mother and Child Health	2014	Enhance food and nutrition security for vulnerable populations
Second National Agricultural Policy	Agricultural policy	Ministry of Agriculture,<br>Ministry of Livestock	2016-2021	• Promote agricultural diversification production and utilisation*<br>• Improve access to seed for biofortified crops*

				• Preservation and utilisation of nutrient-dense foods<br>• Production and utilisation of nutritious food<br>• Nutrition education
Seventh National Development Plan	Investment plan	All sectors	2017-2021	• Improving food and nutrition as a cross-cutting issue<br>• Micronutrient supplementation and fortification*<br>• Revise food and nutrition laws*<br>• Nutrition education and awareness creation<br>• Food and nutrition research promotion
National Food and Nutrition Strategic Plan	Plan	Ministry of Health,<br>Ministry of Education,<br>Ministry of Community Development, Mother and Child Health,<br>Ministry of Agriculture and Livestock,<br>Ministry of Commerce, Trade and Industry	2017-2021	• Prevent child stunting*<br>• Improve food and nutrition security<br>• Improve nutrition education<br>• Control and prevent diet-related NCDs<br>• Enhance coordination of nutrition interventions*<br>• Promote nutritional messages<br>• Strengthen nutrition coordination*
Food and Nutrition Act 2020	Act	National Food and Nutrition Commission	2020	• Realignment of functions for the National Food and Nutrition Commission*<br>• Strengthen and broaden nutrition coordination mandate of the National Food and Nutrition Commission

*Specifically emphasised in the document
